# Association between pharmacotherapy for ADHD in offspring and depression-related specialty care visits by parents with a history of depression

**DOI:** 10.1186/s12888-019-2211-7

**Published:** 2019-07-17

**Authors:** Qi Chen, Henrik Larsson, Catarina Almqvist, Zheng Chang, Paul Lichtenstein, Brian M. D’Onofrio, Jonas F. Ludvigsson

**Affiliations:** 1 Department of Medical Epidemiology and Biostatistics, Karolinska Institutet, Nobels Väg 12A, SE-17177 Stockholm, Sweden; 2 School of Medical Sciences, Örebro University, Örebro, Sweden; 3 Pediatric Allergy and Pulmonology Unit at Astrid Lindgren Children’s Hospital, Karolinska University Hospital, Stockholm, Sweden; 4 Department of Psychological and Brain Sciences, Indiana University, Bloomington, Indiana USA; 5 Department of Pediatrics, Örebro University Hospital, Örebro, Sweden; 6 Division of Epidemiology and Public Health, School of Medicine, University of Nottingham, Clinical Sciences Building 2, City Hospital, Nottingham, UK; 7 Department of Medicine, Columbia University College of Physicians and Surgeons, New York, USA

**Keywords:** ADHD, Pharmacotherapy, Depression, Offspring, Parents

## Abstract

**Background:**

Pharmacotherapy is effective in reducing the core symptoms of attention-deficit/hyperactivity disorder (ADHD). We aimed to investigate the concurrent association between pharmacotherapy for ADHD in offspring and depression-related specialty care visits by the parents with a history of depression.

**Methods:**

Using data from a variety of Swedish national registers, we conducted a cohort study with 8-year follow-up of 5605 parents (3872 mothers and 1733 fathers) who had a history of depression and an offspring diagnosed with ADHD. The hazard rate for parental depression-related specialty care visits during exposed periods when the offspring was on medication for treatment of ADHD was compared with the hazard rate during unexposed periods when the offspring was off medication. Within-individual comparisons were employed to control for time-constant confounding factors.

**Results:**

Among mothers, the crude rates of depression-related specialty care visits during exposed and unexposed periods were 61.33 and 63.95 per 100 person-years, respectively. The corresponding rates among fathers were 49.23 and 54.65 per 100 person-years. When the same parent was compared with him or herself, fathers showed a decreased hazard rate for depression-related visits during exposed periods when the offspring was on medication for treatment of ADHD as compared to unexposed periods (hazard ratio, 0.79 [95% confidence interval, 0.70 to 0.90]). No statistically significant associations were observed in mothers.

**Conclusions:**

Among parents with a history of depression, pharmacotherapy for ADHD in offspring is concurrently associated with a decreased rate of depression-related specialty care visits in fathers but not in mothers. Future research with refined measures of parental depression and other time-varying familial factors is needed to better understand the mechanisms underlying the association.

## Background

Attention-deficit/hyperactivity disorder (ADHD) is a neurodevelopmental disorder affecting both children and adults [1–3]. Inattention, hyperactivity, and impulsivity constitute core symptoms of ADHD [4]. Children and adolescents with ADHD are at increased risk of conduct disorder [5], learning disabilities [5], substance use disorder [6], criminality [7], and injuries [8], which might add to the overall burden on caregivers.

A multi-country online survey conducted among 2326 caregivers of children/adolescents with ADHD showed that the caregivers experienced considerable burden in terms of work, social activity, family life, and parental stress, despite pharmacotherapy received by all children/adolescents during the previous 6 months [9]. In addition, the survey found that ADHD medication adherence was associated with reduced caregiver burden related to work and social activity [9]. One limitation of the survey study was the lack of an optimal proxy for off-medication time.

High caregiver burden has been linked to depression among caregivers of patients with different health problems [10–12]. Previous research found that parents of offspring with ADHD tend to show more depressive symptoms and parental stress than parents of offspring without ADHD [13–15]. While pharmacotherapy is effective in reducing core symptoms of ADHD [16, 17], and associated with decreased risks of adverse health outcomes [7, 8, 18], to date, it remains unclear whether pharmacotherapy for ADHD in offspring may benefit the parents, for example, by mitigating parental depression.

One substantial challenge to such investigation results from the genetic overlap between ADHD and depression. The most up-to-date meta-analysis of genome-wide association studies (GWASs) of ADHD reported a moderate genetic correlation (about 0.4) between ADHD and depression [19]. Because of the genetic sharing between offspring and their parents, offspring who received pharmacotherapy for ADHD might be more likely to have parents with depression than offspring without ADHD, even in the absence of any causal relationship. This is an issue similar to confounding by indication in pharmacoepidemiological studies [20]. Nonetheless, the Swedish national health registers provide medical records of hospital discharge and drug dispensation, enabling us to compare the rate for depression-related specialty care visits by parents during the time periods when their ADHD-affected offspring are on medication with the rate during all other time periods. When comparisons are made within the same parents across different time periods, all time-constant factors, such as genetic makeup and disease severity at baseline, are implicitly cancelled out.

Using data from the Swedish national registers, we conducted a cohort study to investigate the concurrent association between pharmacotherapy for ADHD in offspring and rate of depression-related specialty care visits by the parents with a history of depression.

## Methods

### Data sources and study population

All residents in Sweden are assigned a unique personal identity number that allows for large-scale linkage across the Swedish national registers [21]. The Medical Birth Register contains data on 99% of all births in Sweden since 1973 [22]. The National Patient Register was established in 1964 and covers almost 100% of inpatient care, with information on psychiatric inpatient care being added since 1973 [23]. From 2001 onwards, the register also includes data on outpatient specialty care visits to the public caregivers, whereas data from the private caregivers have been missing. Although we are not aware of any systematic validation for ADHD and depression diagnoses in the National Patient Register, most diagnoses in the register have a positive predictive value of about 85–95% [23]. The Total Population Register contains information on births, deaths, and migrations [24]. Virtually all births and deaths are reported within 30 days of the event. The Multi-Generation Register links individuals born in Sweden since 1932 and registered as living in Sweden since 1961 to their biological parents [25].

By linking these registers, we identified offspring born between January 1, 1992, and December 31, 2003 and later diagnosed with ADHD according to the *International Classification of Disease, 10th, revision* (ICD-10 code: F90). We then identified their parents who were born from January 1, 1964 onwards and had at least one specialty care visit with depression as the primary diagnosis. In the current study, while the offspring defined the exposure (i.e., on- or off-treatment by ADHD medication), the parents constituted the cohorts. In total, we identified 3872 mothers and 1733 fathers fulfilling the inclusion criteria. Mothers and fathers were separately followed up for future depression-related visits from January 1, 2006, the offspring’s 6th birthday, or the first depression-related visit, whichever came last, until death, death of the offspring, the offspring’s 18th birthday, or December 31, 2013, whichever occurred first. All offspring were at least 6 years old when their parents entered the follow-up, because ADHD medications are only recommended for children at least 6 years old in Sweden [26].

### Treatment status by ADHD medication

We retrieved information on dispensed ADHD medications based on the Anatomical Therapeutic Chemical (ATC) codes from the Swedish Prescribed Drug Register [27]. The register contains data on dispensed drugs to the entire Swedish population since July 2005, but does not include over the counter medications or medications administered in hospital. In Sweden, ADHD medications are only available through prescription and, with regards to children, almost exclusively prescribed by child psychiatrists or child neurologists. Information on patient identity is only missing on < 0.3% of all registered prescriptions [27]. In the current study, we identified all dispensing dates of four stimulants (methylphenidate [N06BA04], amfetamine [N06BA01], dexamfetamine [N06BA02], and lisdeamfetamin [N06BA12]) and one non-stimulant (atomoxetine [N06BA09]) for treatment of ADHD. In accordance with prior research [18, 28], we divided the follow-up into time periods when offspring were on and off medication for treatment of ADHD. An offspring was considered on medication during the time interval between two consecutive ADHD medication dispensations no longer than 6 months (183 days) apart. An on-medication period started on the date of first dispensation and ended on the date of last dispensation. The remaining time periods were off-medication periods. A detailed description of the definition of exposed period can be found elsewhere [7]. In the current study, 77.1% of the mothers and 79.2% the fathers were exposed to at least one period when their offspring were on medication for treatment of ADHD.

### Outcome events

The outcome events were defined as depression-related specialty care visits including any hospital admissions or outpatient visits to psychiatric specialists, with depression (ICD-10: F32–33) as the primary diagnosis. Information on dates of the visits was retrieved from the National Patient Register. In one of the sensitivity analyses, depression-related unplanned visits were used as secondary outcome events.

### Covariates

The analyses were adjusted for parental baseline characteristics, including civil status (unmarried, married, divorced, or widowed), employment (yes or no), and highest education achieved (basic education, upper secondary education, or tertiary education), as well as age (in years) and sex (male or female) of the offspring. Information for constructing the covariates was retrieved from the Medical Birth Register [22] and the Longitudinal Integration Database for Health Insurance and Labor Market [29]. Missing data on any covariates were less than 1%.

### Statistical analyses

During follow-up, a parent remained at risk for future outcome event after an event occurred, with follow-up time being reset to baseline. Cox proportional hazards models were used to compare the hazard rate for future outcome event in parents during exposed periods with the hazard rate during unexposed periods. The results were presented as hazard ratios (HRs) and 95% confidence intervals (CIs). The models were automatically adjusted for the selected underlying time scale, which was time since last outcome event [30]. Robust standard errors were calculated to account for non-independence between outcome events within the same parent [30]. The models were adjusted for offspring’s age in years as a categorical time-varying covariate, as well as other measured time-constant covariates. Proportional hazard assumption was verified via visual inspection of the plot of Schoenfeld residuals. The analyses were conducted separately in mothers and fathers. A detailed description of the methodology can be found elsewhere [7].

Stratified Cox proportional hazards models were used for within-individual comparisons, with each parent as a separate stratum. The analyses were implicitly adjusted for time-constant factors and explicitly adjusted for offspring’s age in years as a categorical time-varying covariate.

To test the robustness of our findings, we conducted a series of sensitivity analyses. To minimize bias arising from time-varying factors associated with change in ADHD status of parents themselves or disease status of other family members, we restricted the analyses in three sub-cohorts, namely, ADHD unaffected parents, parents having only one ADHD affected child, and parents whose partner was not affected by depression during follow-up. To test influences from potential exposure misclassification, we repeated the analyses using two alternative definitions of on-medication period. First, we used a 4-month (122 days) cut-off (i.e., an on-medication period was redefined as the time interval between two consecutive ADHD medication dispensations no longer than 4 months apart). Second, we extended the primary definition of on-medication period by 30 days. We also explore the association between pharmacotherapy for ADHD in offspring and the hazard rate for depression-related unplanned visits in depressed parents. This outcome definition was used as a proxy for newly onset depressive episode in prior research [31]. Finally, to further explore the association between parental depression-related visits and ADHD treatment initiation in offspring, we plotted the rates of the visits by mothers and fathers per every 8 weeks during 40 weeks before and 40 weeks after the treatment initiation in offspring. The analyses were restricted to 2589 depressed mothers and 1189 depressed fathers whose offspring were new users of ADHD medications. The new users were individuals with the first registered dispensation of any ADHD medications between January 1, 2007, and December 31, 2012, and thus they were free of ADHD medications for at least 1.5 years, given that the Prescribed Drug Register started in July 1, 2005.

All statistical hypotheses were two-sided with the significance level of .05. SAS software version 9.4 was used for constructing the analytic datasets. Survival package in R software version 3.4 was used for statistical analyses.

### Ethics

This study was approved by the Ethics Review Board in Stockholm, Sweden (diary number 2013/862–31/5). Since this was a registry-based study, informed consent was not required [32].

## Results

Among 3872 mothers (Mean [SD] age at baseline: 36.1 [5.1] years), 4194 and 8784 outcome events occurred during 6838 exposed and 13,735 unexposed person-years, giving crude rates of 61.33 (95% CI, 59.48–63.19) and 63.95 (95% CI, 62.62–65.29) per 100 person-years for the respective periods. Among 1733 fathers (Mean [SD] age at baseline: 37.4 [4.9] years), 1532 and 3187 outcome events occurred during 3112 exposed and 5832 unexposed person-years, giving crude rates of 49.23 (95% CI, 46.76–51.69) and 54.65 (95% CI, 53.75–56.54) per 100 person-years for the respective periods. Other baseline characteristics of parents and their offspring are shown in Table 1.

**Table 1:** Baseline characteristics of parents with a history of depression and their offspring with ADHD

Characteristics of parents	Mothers (*n* = 3872)	Fathers (*n* = 1733)
Age at baseline, No. (%)		
20–29 years	445 (11.5)	125 (7.2)
30–39 years	2351 (60.7)	975 (56.3)
40–49 years	1076 (27.8)	633 (36.5)
Civil status, No. (%)		
Unmarried	1459 (37.7)	736 (42.5)
Married	1406 (36.3)	555 (32.0)
Divorced	984 (25.4)	429 (24.8)
Widowed	13 (0.3)	5 (0.2)
Missing	10 (0.3)	8 (0.5)
Highest education achieved, No. (%)		
Basic education	836 (21.6)	439 (25.3)
Upper secondary education	2187 (56.5)	1064 (61.4)
Tertiary education	847 (21.9)	230 (13.3)
Missing	2 (0.1)	0 (0.0)
Employment, No. (%)		
Employed	2074 (53.6)	1092 (63.0)
Unemployed	1792 (46.3)	634 (36.6)
Missing	6 (0.2)	7 (0.4)
Offspring, No.	4340	1928
Sex of offspring, No. (%)		
Female	1315 (30.3)	604 (31.3)
Male	3025 (69.7)	1324 (68.7)
Age of offspring at baseline, No. (%)		
6–10 years	2419 (55.7)	1091 (56.6)
11–15 years	1605 (37.0)	709 (36.8)
16–17 years	316 (7.3)	128 (6.6)
At least one on-medication period, No. (%)	3344 (77.1)	1527 (79.2)

### Between-individual comparisons

Between-individual comparisons (Table 2) showed no significant association between pharmacotherapy for ADHD in offspring and the hazard rate for depression-related visits in either mothers (HR, 1.00 [95% CI, 0.92–1.09]) or fathers (HR, 0.96 [95% CI, 0.87–1.06]).

**Table 2:** Associations between pharmacotherapy for ADHD in offspring and the hazard rate for depression-related specialty care visits in parents

			Between-individual comparison ^a^	Within-individual comparison ^b^
	Parents, No.	Events, No.	HR (95% CI)	HR (95% CI)
Mothers	3872	12,978	1.00 (0.92–1.09)	0.99 (0.91–1.06)
Fathers	1733	4719	0.96 (0.87–1.06)	0.79 (0.70–0.90)

*HR* Hazard ratio, *CI* confidence interval

^a^Between individual comparisons were adjusted for age of the offspring as a time-varying covariate, as well as measured time-constant covariates, including sex of the offspring, civil status, highest education achieved, and employment status of the parent at baseline

^b^Within individual comparisons were adjusted for age of the offspring as a time-varying covariate

### Within-individual comparisons

When the same parent was compared to him or herself across different time periods (Table 2), fathers showed a decreased hazard rate for depression-related visits during exposed periods as compared to unexposed periods (HR, 0.79 [95% CI, 0.70–0.90]). The association, however, was not statistically significant in mothers (HR, 0.99 [95% CI, 0.91–1.06]).

### Sensitivity analyses

The pattern of results from both between- and within-individual comparisons remained in sensitivity analyses in the three sub-cohorts of depressed parents, using two alternative definitions of on-medication period, or using depression-related unplanned visits as the outcome events (Table 3).

**Table 3:** Sensitivity analyses for associations between pharmacotherapy for ADHD in offspring and the hazard rate for depression-related specialty care visits in parents

	Parents, No.	Events, No.	Between-individual comparison ^a^	Within-individual comparison ^b^
			HR (95% CI)	HR (95% CI)
Parents who were not diagnosed with ADHD or treated with ADHD medication				
Mothers	2806	8869	1.04 (0.95–1.15)	0.97 (0.88–1.06)
Fathers	1237	3313	0.96 (0.85–1.08)	0.79 (0.67–0.93)
Parents who had only one ADHD affected child				
Mothers	3455	10,720	1.01 (0.92–1.12)	0.97 (0.90–1.06)
Fathers	1553	3751	0.95 (0.84–1.07)	0.81 (0.70–0.94)
Parents whose partner were free of depression during follow-up				
Mothers	3716	12,350	1.00 (0.92–1.09)	0.99 (0.92–1.07)
Fathers	1559	4166	0.96 (0.86–1.07)	0.79 (0.69–0.91)
On-medication period redefined by a four-month cut-off				
Mothers	3872	12,978	1.01 (0.93–1.10)	1.01 (0.94–1.08)
Fathers	1733	4719	0.95 (0.86–1.05)	0.78 (0.69–0.88)
Extending on-medication period by 30 days				
Mothers	3872	12,978	1.00 (0.92–1.08)	0.98 (0.91–1.06)
Fathers	1733	4719	0.96 (0.87–1.07)	0.80 (0.70–0.90)
Depression-related unplanned visits as outcome events				
Mothers	1694	1116	1.04 (0.82–1.33)	1.22 (0.86–1.73)
Fathers	886	503	1.02 (0.83–1.25)	0.58 (0.35–0.94)

*HR* Hazard ratio, *CI* confidence interval

^a^Between individual comparisons were adjusted for age of the offspring as a time-varying covariate, as well as measured time-constant covariates, including sex of the offspring, civil status, highest education achieved, and employment status of the parent at baseline

^b^Within individual comparisons were adjusted for age of the offspring as a time-varying covariate

**Fig. 1:**
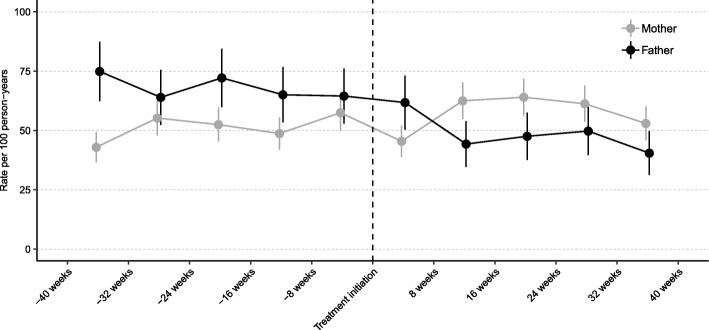
Rate and 95% CI of depression-related visits per every 8 weeks in parents during 40 weeks before and 40 weeks after ADHD treatment initiation in offspring. The analyses were restricted to 2589 mothers and 1189 fathers whose offspring were new users of ADHD medications between January 1, 2007 and December 31, 2012

Finally, despite some fluctuation, the rate of depression-related visits during 40 weeks before ADHD treatment initiation in children appeared to be stable in both mothers and fathers. After treatment initiation, the rate in mothers remained relatively stable; the rate in fathers decreased within 16 weeks, nearly leveled off afterwards until it decreased again after approximately 32 weeks (Fig. 1).

## Discussion

In this large cohort study, we followed up 5605 parents (3872 mothers and 1733 fathers) with a history of depression and an offspring diagnosed with ADHD for 8 years to investigate the concurrent association between pharmacotherapy for ADHD in offspring and rate of depression-related visits in parents.

Between-individual analyses yielded no difference in the hazard rate for depression-related visits in parents during exposed periods when their offspring were on medication for treatment of ADHD as compared to unexposed periods. Nonetheless, the analyses could not take into consideration individual specific baseline severity of depression, and other unmeasured time-constant or time-varying factors. In within-individual analyses, we compared the same parent across time periods when his or her offspring was on and off medication for treatment of ADHD. Only those parents who had at least one outcome event during follow-up and whose offspring had both on-and off-medication periods contributed to the analyses. Within-individual comparisons helped rule out bias arising from time-constant factors, such as genetic makeup and disease severity at baseline, and thus provided more valid estimates than between-individual comparisons. Based on within-individual comparisons, we detected an association between pharmacotherapy for ADHD in offspring and a decreased hazard rate for depression-related visits in fathers. No statistically significant association was observed in mothers. The pattern of results remained in all sensitivity analyses, suggesting the findings were not due to our definitions of study cohort, exposure, or outcome event.

In the current study, we speculate that the change in hazard rate for depression-related visits in parents might to some extent reflect the change in severity of depression during exposed and unexposed periods. Our findings then could be explained by proper management of ADHD in offspring leading to decrease in severity of depression in the fathers. The process might be mediated by father-perceived improvements in children’s ADHD and subsequent stress-relief, as proposed by two prior clinical studies reporting parent-perceived improvements in children’s ADHD-related behavioral symptoms, parenting stress, and depressive symptoms in parents following methylphenidate treatment for ADHD in the children [33, 34]. Alternatively, our findings could be attributed to that while offspring were on medication for treatment of ADHD, their parents were likely to receive interventions aiming at training parents themselves to appropriately handle children’s ADHD-related behaviors, reducing parental stress, and increasing parental confidence [35], which might also lead to improvement in depression. In the current study, we did not observe statistically significant association between pharmacotherapy for ADHD in offspring and depression-related visits by mothers. Nonetheless, this does not necessarily mean that there was no decrease in severity of maternal depression associated with pharmacotherapy for ADHD in the offspring, given the lack of direct measurements for depressive symptoms.

To examine whether the observed associations for fathers were due to reverse causation (i.e., offspring with ADHD might be more likely to be on medication for treatment of ADHD when their parents were less depressed), we plotted the rates of depression-related visits by parents per every 8 weeks during 40 weeks before and 40 weeks after treatment initiation in offspring. We observed no obvious drop in the rate shortly before treatment initiation either in mothers or in fathers, suggesting that treatment initiation in offspring was unlikely triggered by parents being less depressed. Nonetheless, we cannot rule out the possibility that decrease in severity of depression in fathers might help improve their offspring’s compliance to treatment of ADHD. More research is needed to unravel the role of the complex and dynamic parent-offspring interactions in the associations under study.

The main strengths of our study include the large sample and prolonged follow-up, both enhancing the statistical power for detecting small effects. The Multi-Generation Register allowed unambiguous identification of parent-offspring relations. The prospectively collected data on medication dispensations and depression-related specialty care visits precluded recall bias. Furthermore, we employed an innovative within-individual comparison design to control for unmeasured time-constant factors that often threaten the validity of observational studies. In addition, since the exposure and outcome events were separately defined in offspring and their parents, our study was not subject to bias due to confounding by contraindication (i.e., in an observational study, a medication might seem to have a protective effect on an outcome when the outcome is a contraindication for the medication) [36].

We also acknowledge some limitations of the study. First, we are not aware of any validation study on ADHD diagnosis in the National Patient Register. Nevertheless, a prior study of 19,150 twins in Sweden found that 70% twins with an ADHD diagnosis also screened positive for ADHD by their parents [37]. In the current study, over 77% of the children with ADHD were also dispensed ADHD medications, indicating that they were likely to be true ADHD cases. More importantly, it was mainly the parents of these 77% children that contributed information to the within-individual analyses. Second, on-medication periods were defined empirically using medication dispensations. Although used in many studies, the definition might not precisely reflect the actual consumption of ADHD medications by the offspring. The potential misclassification in exposure, however, would tend to bias the estimates towards null and therefore would not be responsible for the observed significant associations in fathers. Indeed, when on-medication period was redefined using two alternative approaches, the results remained similar to the main results. Third, as in many other studies using within-individual comparisons, unmeasured or even unknown time-varying factors could not be handled by the analyses. Although our results restricted to the three sub-cohorts were less likely affected by time-varying factors associated with change in ADHD status of parents themselves or ADHD/depression status of other family members, other comorbidities or life events occurring to the offspring or their parents during follow-up might still bias the estimated associations. Fourth, considering that pharmacotherapy for ADHD is reserved to relatively severe cases in Sweden [38], our findings apply primarily to parents of offspring with ADHD that was severe enough to warrant pharmacotherapy. Consequently, the generalizability of the findings to other populations is unclear but worth further investigation. Fifth, we were not able to take into account the influences of parent-offspring interactions and non-pharmacological treatments received by either offspring or parents on the associations of interest due to the lack of relevant data.

## Conclusions

Among parents with a history of depression, pharmacotherapy for ADHD in offspring is concurrently associated with a decreased rate of depression-related specialty care visits by fathers but not in mothers. Future research with refined measures of parental depression and other time-varying familial factors is needed to better understand the mechanisms underlying the association.

## Data Availability

The data that support the findings of this study contain sensitive personal information and thus are not publicly available as they are subject to secrecy in accordance with the Swedish Public Access to Information and Secrecy Act. Data are however available from the authors upon reasonable request and with permission from the Swedish Central Ethical Review Board (kansli@cepn.se). Requests for data can be made to the Department of Medical Epidemiology and Biostatistics in Karolinska Institutet (internservice@meb.ki.se).

## Abbreviations

ADHD: Attention-deficit/hyperactivity disorder
CI: Confidence interval
GWAS: Genome-wide association study
HR: Hazard ratio
ICD: International Classification of Diseases
